# A Rigid, Anchylosed, Human Skeleton, the Result of Rheumatism

**Published:** 1856-06

**Authors:** H. P. C. Wilson

**Affiliations:** Physician to the Baltimore City and County Alms House; Baltimore


*A Rigid, Anchylosed, Human Skeleton, the result of Rheumatism.* By H. P. C. Wilson, Physician to the Baltimore City and County Alms House.

Among many anomalous cases which have come under my care at the Baltimore City and County Alms House, there is none which seems more worthy of a place in your journal than the subjoined one of anchylosis. The specimen was viewed with great interest at the Pathological Society of Baltimore, before which it was laid; and it becomes my pleasure to place it on record for the benefit of the whole profession.

This case, so far as I am aware, is unparalleled in the extent of ossification. Nor is the anomaly of a completely rigid, ossified human frame more curious than the fact, that this condition was associated with a healthy performance of all the functions of animal life.

The case was under my care for four months previous to death, during which time I obtained the following history:—

E. E. was admitted to the Alms House in May, 1846. Was then 21 years old. Was a native of Germany, and had come to this country some three years previously. Had been married twice. By first husband had two children, both dead. By last husband had one child, still living. Two years before admission, was attacked with rheumatism, with which she suffered uninterruptedly with greater or less severity up to the time she came in. Was a laboring woman, and had to undergo much exposure, and endure many privations to obtain a support for herself and child. Her second husband being dead, and her disease, from having been acute, becoming chronic, so that her joints began to stiffen and incapacitate her for labor, she was compelled to take refuge in the Baltimore City and County Alms House.

When admitted, could help herself in various ways; could feed herself, turn in bed, walk, and perform most of the other motions of which the joints are capable, but most of them with difficulty, and none with the facility belonging to healthy, uncomplicated joints.

She went on from bad to worse; and when I took charge of the hospital, in March, 1855, I found her lying on her back, without the ability to move a single joint in the body, save a very partial motion of the lower jaw, and the costo-vertebral articulations. Her hands were pronated, and her fore-arms flexed upon the arms, and resting upon the upper part of the abdomen. The soles of her feet were applied one to another. Her legs were flexed upon her thighs, and her thighs upon the pelvis. This, I am told, has been her position, from soon after the date of admission until the date of death, a period of nine years, during which time she has remained a rigid, motionless being. Dry gangrene had taken place in many of her fingers and toes; and in this dry parched state were twisted upon themselves in various ways, so as to resemble more the talons of birds of prey, than what they were.

The various functions of animal life were performed well. Slept well; appetite good, circulation, defecation, and urination all good. Was a picture of patience, and seemed not to suffer a great deal, except at certain periods, as during stormy weather. Was fed on liquids, being incapable of mastication.

She died of typhoid fever, supervening on scurvy, July 9th, 1856.

By inspecting the skeleton of the above subject now in my possession, the inter-articular cartilages of the upper and lower jaw will be found completely ossified; with such an amount of bony matter thrown out in and around the joint as to allow but very partial motion. The history of the case substantiated the same during life.

The occipito-atloid articulation is so anchylosed, as to blot out all evidences of a joint having ever existed.

The intervertebral substances are converted into bone, and the spinal column thus changed into a rigid inflexible pillar.

The sterno-clavicular, and scapulo-clavicular articulations are completely obliterated, the three bones being united into one, without the trace of a joint.

The xiphoid and costal cartilages are all changed into bone. Each humerus is anchylosed to its scapula. Each radius and ulna to its humerus, and to each other. Each carpus is as a single bone, firmly anchylosed at the wrist joint. Each metacarpal bone, with its phalanges, is as one firm, inflexible bone. Similar to the wrist, and hand, and fingers, is the anchylosed condition of the ankle, foot, and toes; and as for the radius, ulna, and humerus, so for the tibia, fibula and femur.

No joint in the body then was capable of the slightest motion, save the two above mentioned, and in these, motion was but very partial. The bones were exceeding light, not weighing one third as much as their counterparts in the healthy subject. The earthy matter was apparently wholly removed from the cancellated structure.

It is to be regretted that circumstances prevented me from making a careful autopsy of this subject. No doubt ossific matter would have been found extensively deposited in and about various organs, (especially the circulatory system) to add additional wonder to this truly anomalous case.

*Baltimore, April 14th,* 1856.

